# Supplementary material for: The Importance of Energy Theory in Shaping Elevational Species Richness Patterns in Plants
Source: Biology (Basel). 2022 May 26;11(6):819. doi: 10.3390/biology11060819 (PMC9219821; doi:10.3390/biology11060819)
Supplement: Supplementary file 1 [file biology-11-00819-s001.zip › biology-1695634-supplementary.pdf]

Figure S1. Elevational species richness patterns of plants collected for 22 mountains. Species richness was linked with elevation using linear, quadratic or cubed models. The red square represents trees; orange triple represents shrubs; purple circles represent herbs, and green diamonds represents ferns.

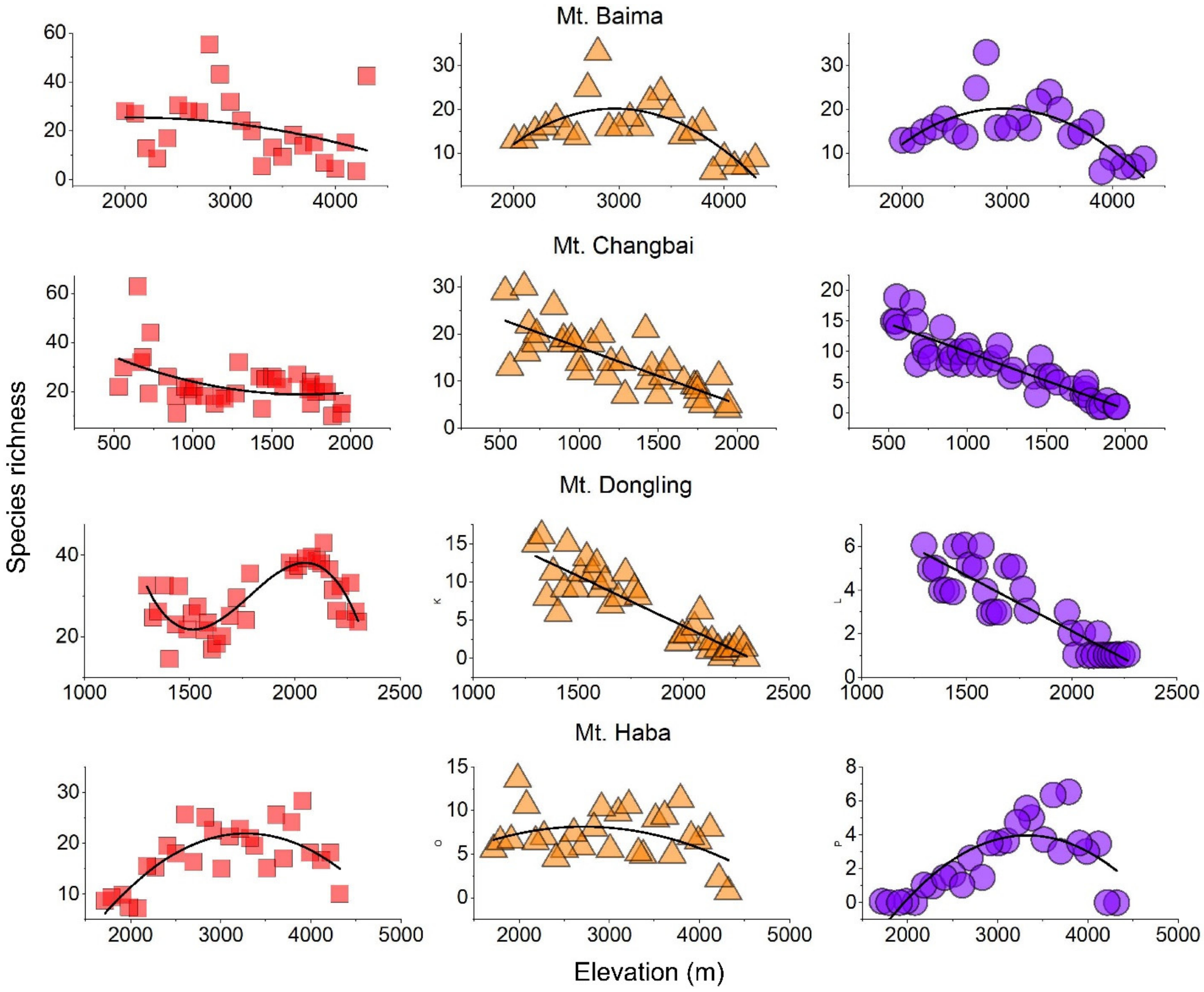

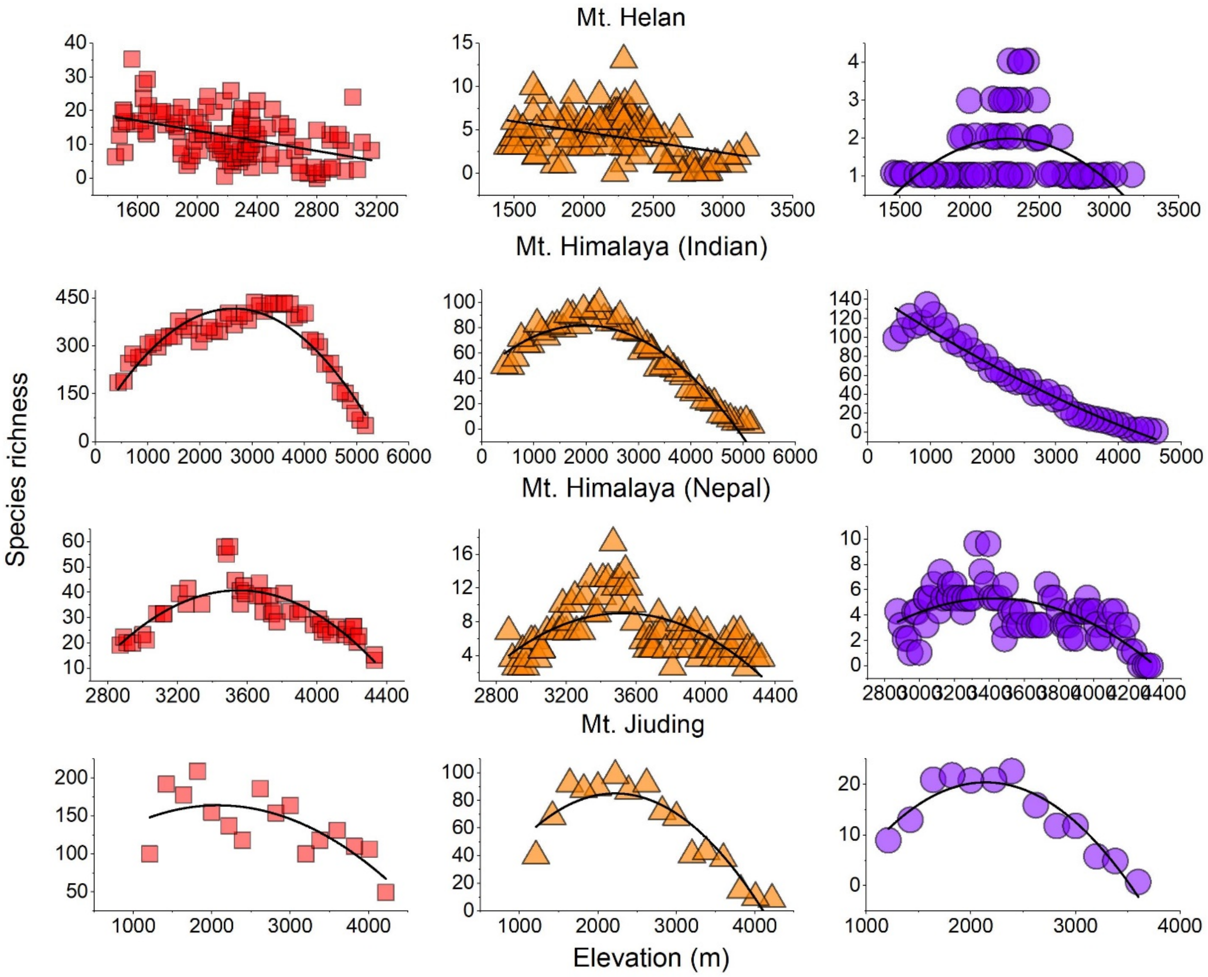

Mt. Helan
Mt. Himalaya (Indian)
Mt. Himalaya (Nepal)
Mt. Jiuding
Species richness
Elevation (m)

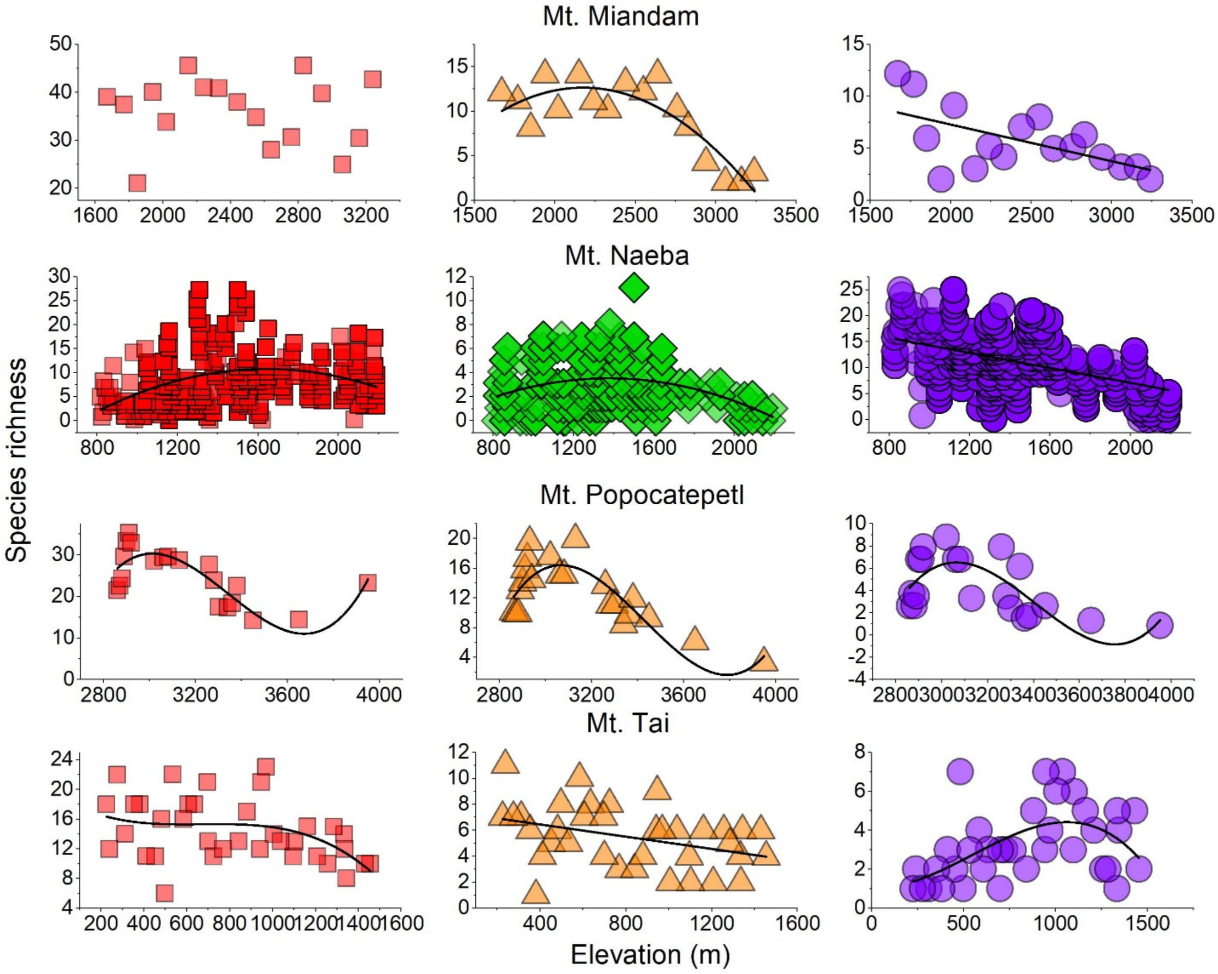

Mt. Miandam
Mt. Naeba
Mt. Popocatepetl
Mt. Tai
Species richness
Elevation (m)

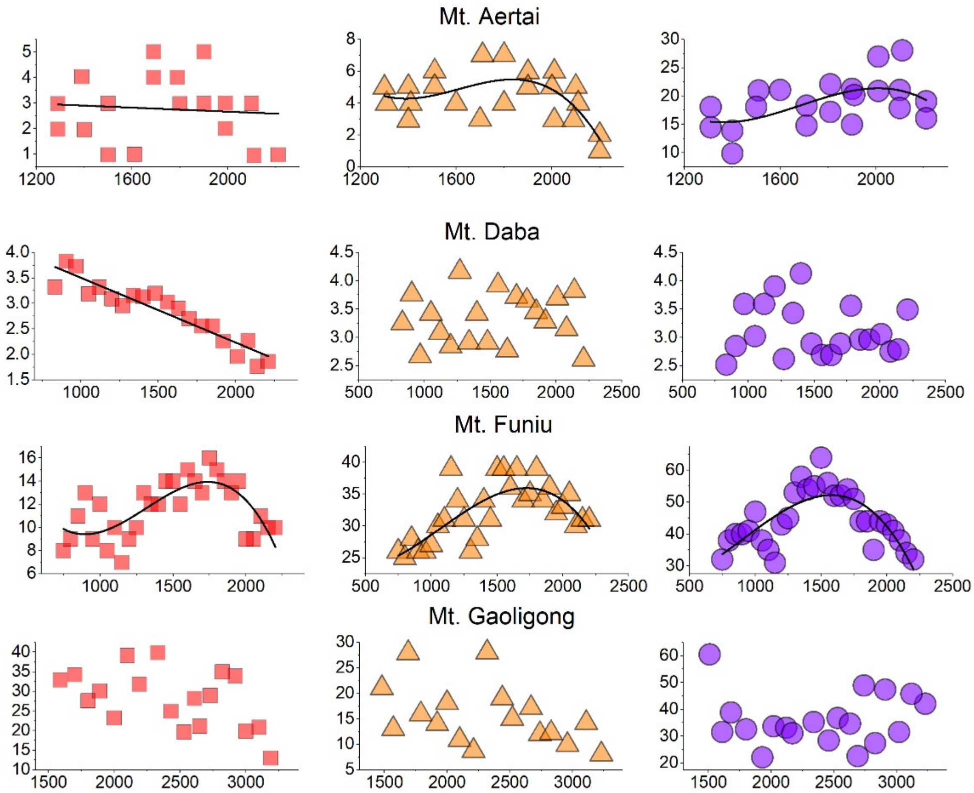

Mt. Aertai
Mt. Daba
Mt. Funiu
Mt. Gaoligong

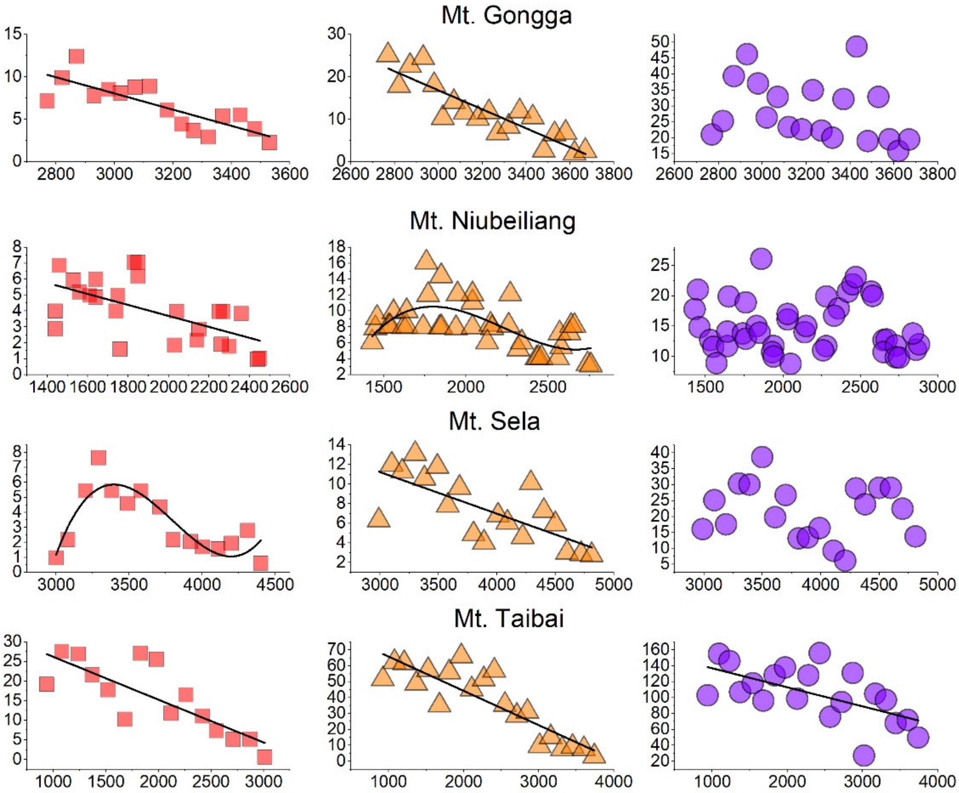
Mt. Gongga
0 5 10 15
2800 3000 3200 3400 3600
0 10 20 30
2600 2800 3000 3200 3400 3600 3800
15 20 25 30 35 40 45 50
2600 2800 3000 3200 3400 3600 3800
Mt. Niubeiliang
0 1 2 3 4 5 6 7 8
1400 1600 1800 2000 2200 2400 2600
2 4 6 8 10 12 14 16 18
1500 2000 2500
10 15 20 25
1500 2000 2500 3000
Mt. Sela
0 1 2 3 4 5 6 7 8
3000 3500 4000 4500
2 4 6 8 10 12 14
3000 3500 4000 4500 5000
5 10 15 20 25 30 35 40
3000 3500 4000 4500 5000
Mt. Taibai
0 5 10 15 20 25 30
1000 1500 2000 2500 3000
0 10 20 30 40 50 60 70
1000 2000 3000 4000
20 40 60 80 100 120 140 160
1000 2000 3000 4000

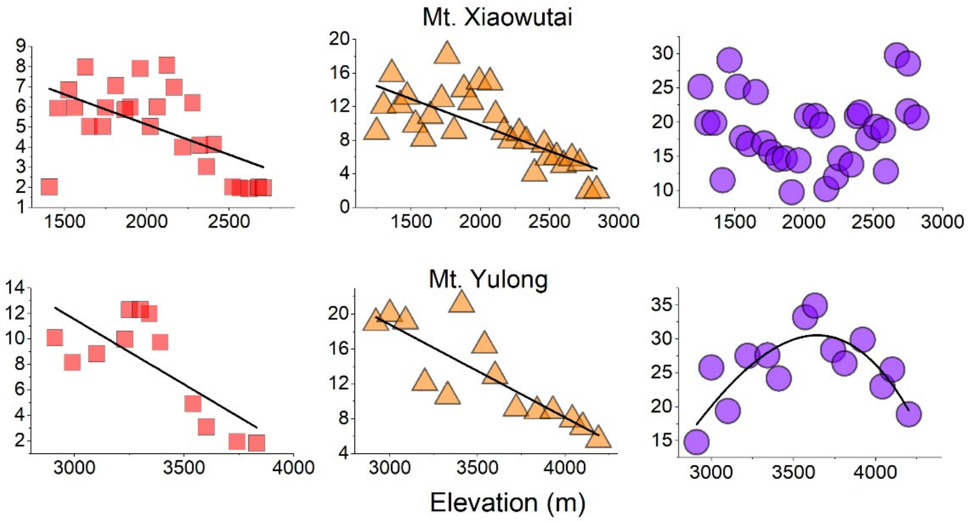

Mt. Xiaowutai
Mt. Yulong
Elevation (m)

Table S1. Relationships between the candidate variables and the elevational species richness patterns of plants with different growth forms. We fitted single variable GLMs of elevational species richness patterns as a linear and unimodal function of each candidate factor. The performance of each model is ranked accounting to the Akaike information criterion (AIC). The highest two performance models are highlighted in deep grey, the third and fourth best models are highlighted with light grey. Area: mountain area of each elevation band; ISO: Isothermality; PS: Precipitation Seasonality; PWQ: Precipitation of Wettest Quarter; PDQ: Precipitation of Driest Quarter; Max: annual maximum temperature; Min: annual minimum temperature; MAT: mean annual temperature; MAP: Mean Annual Precipitation; Vapor : water vapor pressure; AET: actual evapotranspiration; blank represent no significant elevational pattern.

		**Area**	**Climatic variability**		**Tolerance**				**Energy**			
Mt.	Growth form	Area	ISO	PS	PWQ	PDQ	Max	Min	MAT	AET	Vapor	MAP
Aertai	Herb		120.08	**119.66**		**120.07**	**119.91**		**119.86**			120.26
	Shrub	**78.16**										
	Tree	**57.14**										
Baima	Herb		**186.82**									
	Shrub					**153.91**	**145.70**	**146.59**	**145.46**			

	Tree	114.17	**105.58**	127.38	120.81	132.03	**111.30**	**110.29**	112.43	116.43	117.23	**106.95**
Changbai	Herb	**270.28**	272.64		270.71		270.62	**270.28**	272.58	272.64	**269.54**	**265.92**
	Shrub	205.40	206.92	208.87	214.05	219.28	**202.06**	206.61	**202.37**	**202.29**	**201.97**	208.90
	Tree	199.84	186.08	207.63	212.71	221.11	**181.24**	191.96	**180.83**	**181.79**	**179.59**	186.64
Daba	Herb											
	Shrub											
	Tree		7.61	-2.48	-0.83	-3.65	-7.07	-7.08	-7.09	-6.97	-2.37	-7.22
Dongling	Herb	**223.93**		233.83	**228.88**	234.48	233.57	**233.32**	233.80	233.78	233.67	**233.26**
	Shrub	181.16	209.41	161.43	163.43	**157.23**	157.97	157.86	157.74	**157.69**	**157.73**	**157.63**
	Tree	106.276	135.32	87.20	**83.08**	86.55	85.87	**85.67**	**85.71**	86.04	85.89	**85.26**
Funiu	Herb	**196.02**		214.01	**212.69**		215.40	**205.31**	**213.71**			214.48
	Shrub	166.34			179.58	177.48	159.87	**154.97**	**158.06**	163.12		**158.58**
	Tree	131.51		137.36	138.68	136.96	132.19	**125.73**	**129.73**	134.62	**130.52**	130.50
Gaoligong	Herb											
	Shrub											
	Tree	121.62	**120.93**	122.34	**119.41**	122.16	121.18	**120.88**	121.39	121.55	121.59	**121.05**
Gongga	Herb											

	Shrub	122.14	**101.08**	105.21		106.60	102.19	102.48	**101.18**	**102.16**	**101.69**	102.40
	Tree	75.62	66.14	**65.34**		**65.44**	**65.78**	65.80	66.05	65.95	66.48	**65.79**
Haba	Herb	168.11	164.43	159.55		159.57	159.33	**157.66**	**158.31**	158.43	**157.44**	**154.45**
	Shrub											
	Tree	107.37	104.64	104.23	106.96	**85.93**	97.46	**96.13**	**97.41**	101.24	102.41	**93.09**
Helan	Herb	**732.27**	732.57	732.72	**728.33**		**732.53**	733.56	732.58	734.56	**731.76**	733.82
	Shrub	496.97	484.01	505.90	484.36	512.29	**479.60**	**480.82**	479.41		**479.63**	485.10
	Tree					**195.14**				**194.43**		
Himalaya	Herb	547.07	578.05		**537.44**	556.62	**531.62**	**502.97**	**517.77**			576.27
(Indian)	Shrub	418.61	395.54	436.38	375.18	456.77	**340.58**	360.81	347.58	**303.76**	**301.53**	**319.10**
	Tree			385.38	**273.00**	429.53	**301.70**	**301.73**	304.39	310.55	**303.88**	315.12
Himalaya	Herb	352.14	338.03		**313.80**	337.99	**309.61**	333.60	321.86	**316.69**	328.55	**321.14**
(Nepal)	Shrub	397.45	383.04	386.33	**367.27**	384.80	**359.33**	382.15	**372.83**		378.11	**371.53**
	Tree	298.11	289.10	292.89	**280.56**	**282.70**	287.02	284.43	283.60	**281.08**	282.73	**279.89**
Jiuding	Herb		163.40				**160.12**	**160.74**	**160.28**	**159.49**	161.22	

	Shrub	141.04	**121.78**				134.29	**134.13**	134.38	**128.81**	**126.95**	155.17
	Tree	71.23	**62.60**				**68.31**	72.06	**69.64**	**62.73**	70.34	72.40
Miandam	Herb											
	Shrub	**78.86**	**77.40**		92.21	92.07	79.89	**78.90**	79.15	**79.14**	81.40	**78.86**
	Tree	85.86	84.00	**81.91**		84.17	**83.35**	83.65	**83.58**	83.72	**83.16**	85.86
Naeba	Fern		**1571.99**		**1568.25**							**1563.09**
	Herb	4898.50		**4903.03**		**4908.47**	4911.18	4910.77	4911.32	**4909.84**	**4908.94**	
	Tree	6470.33	**6356.69**	6544.46	**6435.67**	6624.18	**6445.89**	6447.73	6446.07	6451.47	6458.87	**6435.83**
Niubeiliang	Herb											
	Shrub	**177.68**	**177.51**	**179.91**	187.27	194.71	181.60	181.83	180.89	**180.72**	184.70	181.65
	Tree	**97.54**	**97.81**	106.33	101.17	105.50	97.86	97.90	**97.77**	**97.84**	98.30	97.93
Popocatepetl	Herb	**124.27**	**119.26**	**118.58**	125.10	123.91	125.94	126.60	126.09	**122.42**	125.88	125.90
	Shrub	**114.64**	**113.94**	**104.66**	115.82	116.06	114.97	115.86	115.50	**114.53**	116.27	114.63
	Tree	**91.22**	**89.34**	**87.22**	**91.92**	92.63	94.26	94.76	94.37	92.61	93.55	93.65
Sela	Herb											

	Shrub	96.51	**90.96**	93.58	96.52	92.18	91.18	**90.77**	**90.46**	91.34	**90.29**	91.02
	Tree	65.05			**50.51**	65.68	**63.89**	64.72	63.91	**63.29**	**53.28**	64.14
Tai	Herb			**202.95**	**205.34**							
	Shrub	**268.32**		274.94	272.74		**272.26**	273.09	272.43	272.56	**272.34**	**272.10**
	Tree	142.56	186.82		144.08	146.99	**140.50**	140.84	**140.53**	**140.51**	**140.57**	140.71
Taibai	Herb	198.58	202.34	198.77	**194.15**	**194.05**	**194.16**	193.83	194.36	194.44	197.95	**193.99**
	Shrub	174.29	178.56	174.30	152.01	158.15	**148.90**	149.26	**149.09**	**149.18**	161.22	**149.20**
	Tree				99.91	**97.57**	**98.23**	98.29	**98.20**	98.38	**96.81**	98.32
Xiaowutai	Herb											
	Shrub	150.83	153.46	146.64	**138.88**	**136.78**	**139.30**	143.03	**138.67**	140.41	139.95	140.31
	Tree		103.35	100.40	93.19	98.28	**90.44**	92.18	**89.12**	**90.45**	90.83	**90.40**
Yulong	Herb											
	Shrub	75.28	76.91	91.22	87.28	87.43	**74.04**	**74.12**	74.95	**74.76**	76.32	**74.15**
	Tree	**54.91**	**53.26**	67.03	69.64	65.25	55.45	**54.84**	55.09	55.41	**51.84**	54.93

Table S2. Best fitting multiple and single factor model for each plant growth forms of elevational species richness pattern. The factors showing the best performance in each hypothesis group were selected as the best candidate factor combination; Adjusted R-squared are given for the best models remove collinearity.

Mt.	Growth form	Best model	Models remove collinearity	Adjusted R-squared
Aertai	Herb	PS+Min+AET	PS	0.19
	Shrub	Area+Min+MAP	Area	0.29
	Tree	Area	Area	0.39
Baima	Herb	ISO	ISO	0.35
	Shrub	Max+MAT	MAT	0.47
	Tree	Area+ISO+Max+MAP	ISO	0.70
Changbai	Herb	Area+ISO+Min+MAP	MAP	0.17
	Shrub	Area+ISO+Max+Vapor	Vapor	0.62
	Tree	Area+ISO+Max+Vapor	Vapor	0.81
Daba	Herb			0
	Shrub			0
	Tree	PS+Max+MAT	MAT	0.91
Dongling	Herb	Area+PS+PWQ+MAP	Area	0.43
	Shrub	Area+PS+PDQ+MAP	PDQ	0.80

	Tree	Area+PS+PWQ+MAP	MAP	0.80
Funiu	Herb	Area+PS+Min+Vapor	Min	0.38
	Shrub	Area+ISO+Min+Vapor	Min	0.56
	Tree	Area+ISO+Min+Vapor	Min	0.40
Gaoligong	Herb			0
	Shrub			0
	Tree	Area+ISO+PWQ+MAT		0.21
Gongga	Herb			0
	Shrub	Area+ISO+Max+Vapor	ISO	0.80
	Tree	Area+PS+PDW+Vapor	PS	0.65
Haba	Herb	Area+PS+Max+MAP	MAP	0.58
	Shrub			0
	Tree	Area+PS+PDQ+MAP	PDQ	0.72
Helan	Herb	Area+ISO+PWQ+Vapor	PWQ	0.2432
	Shrub	Area+ISO+Max+MAT	MAT	0.3062
	Tree	PDQ+AET	PDQ+AET	0.1366
Himalaya (Indian)	Herb	Area+ISO+Min+MAT	Min+Area	0.9189
	Shrub	Area+ISO+Max+Vapor	Vapor+Max	0.9731
	Tree	PS+PWQ+Vapor	PWQ	0.9804

Himalaya (Nepal)	Herb	Area+ISO+Max+AET	Area+PWQ	0.7345
	Shrub	Area+ISO+Max+MAP	Area+Max	0.4876
	Tree	Area+ISO+PWQ+MAP	Area+PWQ	0.4082
Jiuding	Herb	ISO+Max+AET	AET	0.473
	Shrub	Area+ISO+Min+Vapor	ISO	0.9113
	Tree	Area+ISO+Max+AET	Area+ISO	0.9315
Miandam	Herb			0
	Shrub	Area+ISO+Min+AET	ISO	0.7598
	Tree	Area+PS+Max+Vapor	Vapor+PS	0.4192
Naeba	Fern	ISO+PWQ+MAP	MAP	0.07994
	Herb	Area+ISO+PDQ+Vapor	Area	0.08423
	Tree	Area+ISO+PWQ+MAP	ISO	0.2595
Niubeiliang	Herb			0
	Shrub	Area+ISO+Max+AET	ISO	0.38
	Tree	Area+ISO+Max+Vapor	Area	0.34
Popocatepetl	Herb	Area+PS+PDQ+AET	PS	0.539
	Shrub	Area+PS+Max+AET	PS	0.6773
	Tree	Area+PS+PWQ+AET	PS	0.4237
Sela	Herb			0

	Shrub	Area+ISO+Min+MAP	MAP	0.50
	Tree	Area+PWQ+MAP	PWQ	0.71
Tai	Herb	PS+PWQ	PS	0.16
	Shrub	Area+Max+MAP	Area	0.19
	Tree	Area+Max+MAT	AET	0.24
Taibai	Herb	Area+PS+Min+MAT	Min	0.34
	Shrub	Area+PS+Max+Vapor	Max	0.83
	Tree	PDQ+MAP	MAP	0.65
Xiaowutai	Herb			0
	Shrub	Area+PS+PDQ+Vapor	PDQ	0.68
	Tree	PS+Max+Vapor	Vapor	0.65
Yulong	Herb			0
	Shrub	Area+ISO+Max+MAT	Max	0.69
	Tree	Area+ISO+Min+MAP	MAP	0.32

Table S3. Pearson correlations coefficient of all candidate factors for model selection of each elevational richness pattern. See Table 2 for abbreviations.

Mt. Aertai-Tree											
	Area	ISO	PS	PWQ	PDQ	Max	Min	Vapor	AET	MAP	MAT
Area	1.00	-0.17	0.96	0.99	0.81	-0.97	-0.98	-0.96	-0.98	-0.99	-0.67
ISO	-0.17	1.00	-0.39	-0.18	-0.47	0.03	0.30	-0.05	0.28	0.19	-0.16
PS	0.96	-0.39	1.00	0.95	0.82	-0.91	-0.99	-0.89	-0.99	-0.98	-0.64
PWQ	0.99	-0.18	0.95	1.00	0.84	-0.95	-0.98	-0.95	-0.98	-0.98	-0.61
PDQ	0.81	-0.47	0.82	0.84	1.00	-0.64	-0.83	-0.66	-0.82	-0.77	-0.15
Max	-0.97	0.03	-0.91	-0.95	-0.64	1.00	0.94	0.99	0.95	0.98	0.80
Min	-0.98	0.30	-0.99	-0.98	-0.83	0.94	1.00	0.93	1.00	0.99	0.64
Vapor	-0.96	-0.05	-0.89	-0.95	-0.66	0.99	0.93	1.00	0.94	0.97	0.78
AET	-0.98	0.28	-0.99	-0.98	-0.82	0.95	1.00	0.94	1.00	0.99	0.66
MAP	-0.99	0.19	-0.98	-0.98	-0.77	0.98	0.99	0.97	0.99	1.00	0.72
MAT	-0.67	-0.16	-0.64	-0.61	-0.15	0.80	0.64	0.78	0.66	0.72	1.00

Mt. Aertai-Shrub											
	Area	ISO	PS	PWQ	PDQ	Max	Min	Vapor	AET	MAP	MAT
Area	1.00	0.19	0.31	-0.24	-0.48	-0.27	-0.60	-0.29	-0.42	-0.67	-0.38
ISO	0.19	1.00	0.99	0.85	-0.95	-0.99	-0.89	-0.99	-0.97	-0.75	-0.98
PS	0.31	0.99	1.00	0.81	-0.97	-1.00	-0.94	-1.00	-0.99	-0.78	-0.99
PWQ	-0.24	0.85	0.81	1.00	-0.65	-0.82	-0.57	-0.81	-0.71	-0.28	-0.76
PDQ	-0.48	-0.95	-0.97	-0.65	1.00	0.96	0.99	0.97	0.99	0.90	0.99
Max	-0.27	-0.99	-1.00	-0.82	0.96	1.00	0.93	1.00	0.98	0.77	0.99
Min	-0.60	-0.89	-0.94	-0.57	0.99	0.93	1.00	0.94	0.98	0.91	0.96
Vapor	-0.29	-0.99	-1.00	-0.81	0.97	1.00	0.94	1.00	0.99	0.78	1.00
AET	-0.42	-0.97	-0.99	-0.71	0.99	0.98	0.98	0.99	1.00	0.86	1.00
MAP	-0.67	-0.75	-0.78	-0.28	0.90	0.77	0.91	0.78	0.86	1.00	0.83
MAT	-0.38	-0.98	-0.99	-0.76	0.99	0.99	0.96	1.00	1.00	0.83	1.00

Mt. Aertai-Herb											
	Area	ISO	PS	PWQ	PDQ	Max	Min	Vapor	AET	MAP	MAT
Area	1.00	0.25	0.35	-0.23	-0.52	-0.33	-0.67	-0.35	-0.58	-0.73	-0.44
ISO	0.25	1.00	0.98	0.82	-0.94	-0.99	-0.88	-0.99	-0.92	-0.75	-0.98
PS	0.35	0.98	1.00	0.76	-0.97	-1.00	-0.93	-1.00	-0.95	-0.78	-0.99
PWQ	-0.23	0.82	0.76	1.00	-0.59	-0.78	-0.50	-0.77	-0.55	-0.25	-0.71
PDQ	-0.52	-0.94	-0.97	-0.59	1.00	0.96	0.97	0.97	0.99	0.90	0.98
Max	-0.33	-0.99	-1.00	-0.78	0.96	1.00	0.92	1.00	0.95	0.78	0.99
Min	-0.67	-0.88	-0.93	-0.50	0.97	0.92	1.00	0.93	0.99	0.93	0.96
Vapor	-0.35	-0.99	-1.00	-0.77	0.97	1.00	0.93	1.00	0.95	0.79	0.99
AET	-0.58	-0.92	-0.95	-0.55	0.99	0.95	0.99	0.95	1.00	0.94	0.98
MAP	-0.73	-0.75	-0.78	-0.25	0.90	0.78	0.93	0.79	0.94	1.00	0.84
MAT	-0.44	-0.98	-0.99	-0.71	0.98	0.99	0.96	0.99	0.98	0.84	1.00

Mt. Changbai-Tree											
	Area	ISO	Map	Mat	Max	Min	Vapor	AET	PWQ	PDQ	PS
Area	1.00	-0.83	-0.90	0.95	0.95	0.94	0.94	0.95	-0.90	-0.86	0.98
ISO	-0.83	1.00	0.82	-0.93	-0.95	-0.85	-0.95	-0.93	0.69	0.54	-0.83
Map	-0.90	0.82	1.00	-0.96	-0.94	-0.99	-0.95	-0.97	0.97	0.89	-0.86
Mat	0.95	-0.93	-0.96	1.00	1.00	0.98	1.00	1.00	-0.90	-0.80	0.93
Max	0.95	-0.95	-0.94	1.00	1.00	0.96	1.00	1.00	-0.88	-0.77	0.93
Min	0.94	-0.85	-0.99	0.98	0.96	1.00	0.97	0.98	-0.96	-0.89	0.91
Vapor	0.94	-0.95	-0.95	1.00	1.00	0.97	1.00	1.00	-0.88	-0.77	0.93
AET	0.95	-0.93	-0.97	1.00	1.00	0.98	1.00	1.00	-0.91	-0.81	0.93
PWQ	-0.90	0.69	0.97	-0.90	-0.88	-0.96	-0.88	-0.91	1.00	0.97	-0.85
PDQ	-0.86	0.54	0.89	-0.80	-0.77	-0.89	-0.77	-0.81	0.97	1.00	-0.82
PS	0.98	-0.83	-0.86	0.93	0.93	0.91	0.93	0.93	-0.85	-0.82	1.00

Mt. Changbai-Shrub, Herb											
	Area	ISO	Map	Mat	Max	Min	Vapor	AET	PWQ	PDQ	PS
Area	1.00	-0.85	-0.90	0.96	0.96	0.94	0.96	0.96	-0.89	-0.86	0.98
ISO	-0.85	1.00	0.80	-0.93	-0.94	-0.84	-0.94	-0.92	0.69	0.57	-0.84
Map	-0.90	0.80	1.00	-0.96	-0.94	-0.99	-0.94	-0.96	0.98	0.92	-0.85
Mat	0.96	-0.93	-0.96	1.00	1.00	0.98	1.00	1.00	-0.91	-0.83	0.93
Max	0.96	-0.94	-0.94	1.00	1.00	0.96	1.00	0.99	-0.88	-0.80	0.93
Min	0.94	-0.84	-0.99	0.98	0.96	1.00	0.96	0.98	-0.96	-0.91	0.90
Vapor	0.96	-0.94	-0.94	1.00	1.00	0.96	1.00	1.00	-0.89	-0.80	0.93
AET	0.96	-0.92	-0.96	1.00	0.99	0.98	1.00	1.00	-0.92	-0.84	0.93
PWQ	-0.89	0.69	0.98	-0.91	-0.88	-0.96	-0.89	-0.92	1.00	0.97	-0.83
PDQ	-0.86	0.57	0.92	-0.83	-0.80	-0.91	-0.80	-0.84	0.97	1.00	-0.82
PS	0.98	-0.84	-0.85	0.93	0.93	0.90	0.93	0.93	-0.83	-0.82	1.00

Mt. Daba-Tree, Shrub, Herb

	Area	ISO	PS	PWQ	PDQ	Max	Min	Vapor	AET	MAP	MAT
Area	1.00	0.50	-0.94	-1.00	0.99	1.00	-1.00	-1.00	-1.00	-1.00	0.99
ISO	0.50	1.00	-0.40	-0.55	0.51	0.50	-0.48	-0.47	-0.52	-0.53	0.43
PS	-0.94	-0.40	1.00	0.92	-0.97	-0.94	0.94	0.93	0.93	0.93	-0.96
PWQ	-1.00	-0.55	0.92	1.00	-0.99	-1.00	0.99	0.99	1.00	1.00	-0.98
PDQ	0.99	0.51	-0.97	-0.99	1.00	0.99	-0.99	-0.99	-0.99	-0.99	0.99
Max	1.00	0.50	-0.94	-1.00	0.99	1.00	-1.00	-1.00	-1.00	-1.00	0.99
Min	-1.00	-0.48	0.94	0.99	-0.99	-1.00	1.00	1.00	1.00	1.00	-0.99
Vapor	-1.00	-0.47	0.93	0.99	-0.99	-1.00	1.00	1.00	1.00	1.00	-0.99
AET	-1.00	-0.52	0.93	1.00	-0.99	-1.00	1.00	1.00	1.00	1.00	-0.99
MAP	-1.00	-0.53	0.93	1.00	-0.99	-1.00	1.00	1.00	1.00	1.00	-0.99
MAT	0.99	0.43	-0.96	-0.98	0.99	0.99	-0.99	-0.99	-0.99	-0.99	1.00

Mt. Dongling-Tree

	Area	ISO	Map	Mat	Max	Min	Vapor	AET	PWQ	PDQ	PS
Area	1.00	-0.39	-0.77	0.70	0.73	0.66	0.69	0.71	-0.85	-0.76	0.70
ISO	-0.39	1.00	-0.24	0.34	0.30	0.39	0.35	0.32	-0.06	-0.24	0.33
Map	-0.77	-0.24	1.00	-0.99	-1.00	-0.98	-0.99	-0.99	0.96	0.99	-0.99
Mat	0.70	0.34	-0.99	1.00	1.00	1.00	1.00	1.00	-0.94	-0.99	1.00
Max	0.73	0.30	-1.00	1.00	1.00	0.99	1.00	1.00	-0.95	-0.99	1.00
Min	0.66	0.39	-0.98	1.00	0.99	1.00	1.00	1.00	-0.92	-0.98	0.99
Vapor	0.69	0.35	-0.99	1.00	1.00	1.00	1.00	1.00	-0.94	-0.99	1.00
AET	0.71	0.32	-0.99	1.00	1.00	1.00	1.00	1.00	-0.95	-0.99	0.99
PWQ	-0.85	-0.06	0.96	-0.94	-0.95	-0.92	-0.94	-0.95	1.00	0.97	-0.94
PDQ	-0.76	-0.24	0.99	-0.99	-0.99	-0.98	-0.99	-0.99	0.97	1.00	-0.99
PS	0.70	0.33	-0.99	1.00	1.00	0.99	1.00	0.99	-0.94	-0.99	1.00

Mt. Dongling-Shrub, Herb											
	Area	ISO	Map	Mat	Max	Min	Vapor	AET	PWQ	PDQ	PS
Area	1.00	-0.43	-0.80	0.73	0.76	0.69	0.72	0.74	-0.87	-0.79	0.74
ISO	-0.43	1.00	-0.16	0.27	0.22	0.32	0.27	0.24	0.03	-0.15	0.25
Map	-0.80	-0.16	1.00	-0.99	-1.00	-0.98	-0.99	-0.99	0.96	0.99	-0.99
Mat	0.73	0.27	-0.99	1.00	1.00	1.00	1.00	1.00	-0.94	-0.99	1.00
Max	0.76	0.22	-1.00	1.00	1.00	0.99	1.00	1.00	-0.95	-0.99	1.00
Min	0.69	0.32	-0.98	1.00	0.99	1.00	1.00	1.00	-0.92	-0.98	0.99
Vapor	0.72	0.27	-0.99	1.00	1.00	1.00	1.00	1.00	-0.93	-0.99	1.00
AET	0.74	0.24	-0.99	1.00	1.00	1.00	1.00	1.00	-0.95	-0.99	0.99
PWQ	-0.87	0.03	0.96	-0.94	-0.95	-0.92	-0.93	-0.95	1.00	0.97	-0.94
PDQ	-0.79	-0.15	0.99	-0.99	-0.99	-0.98	-0.99	-0.99	0.97	1.00	-0.99
PS	0.74	0.25	-0.99	1.00	1.00	0.99	1.00	0.99	-0.94	-0.99	1.00

Mt. Funiu-Tree, Shrub, Herb											
	Area	ISO	PS	PWQ	PDQ	Max	Min	Vapor	AET	MAP	MAT
Area	1.00	-0.94	-0.95	-0.07	0.63	0.82	-0.91	-0.70	-0.86	-0.95	0.87
ISO	-0.94	1.00	0.96	0.12	-0.62	-0.82	0.94	0.77	0.89	0.95	-0.82
PS	-0.95	0.96	1.00	-0.04	-0.52	-0.76	0.98	0.84	0.95	0.99	-0.77
PWQ	-0.07	0.12	-0.04	1.00	-0.81	-0.60	-0.20	-0.50	-0.31	-0.15	-0.49
PDQ	0.63	-0.62	-0.52	-0.81	1.00	0.93	-0.36	0.01	-0.24	-0.42	0.91
Max	0.82	-0.82	-0.76	-0.60	0.93	1.00	-0.64	-0.31	-0.54	-0.69	0.95
Min	-0.91	0.94	0.98	-0.20	-0.36	-0.64	1.00	0.93	0.99	0.99	-0.65
Vapor	-0.70	0.77	0.84	-0.50	0.01	-0.31	0.93	1.00	0.96	0.88	-0.32
AET	-0.86	0.89	0.95	-0.31	-0.24	-0.54	0.99	0.96	1.00	0.98	-0.55
MAP	-0.95	0.95	0.99	-0.15	-0.42	-0.69	0.99	0.88	0.98	1.00	-0.72
MAT	0.87	-0.82	-0.77	-0.49	0.91	0.95	-0.65	-0.32	-0.55	-0.72	1.00

Mt. Gaoligong-Tree											
	Area	ISO	PS	PWQ	PDQ	Max	Min	Vapor	AET	MAP	MAT
Area	1.00	-0.84	-0.94	-0.99	-0.80	-0.99	-1.00	-1.00	-1.00	-1.00	-0.99
ISO	-0.84	1.00	0.97	0.80	0.86	0.77	0.85	0.86	0.83	0.83	0.81
PS	-0.94	0.97	1.00	0.91	0.88	0.89	0.94	0.95	0.93	0.93	0.92
PWQ	-0.99	0.80	0.91	1.00	0.72	1.00	0.99	0.98	0.99	1.00	0.99
PDQ	-0.80	0.86	0.88	0.72	1.00	0.73	0.81	0.83	0.79	0.78	0.78
Max	-0.99	0.77	0.89	1.00	0.73	1.00	0.99	0.98	0.99	0.99	1.00
Min	-1.00	0.85	0.94	0.99	0.81	0.99	1.00	1.00	1.00	1.00	1.00
Vapor	-1.00	0.86	0.95	0.98	0.83	0.98	1.00	1.00	1.00	1.00	0.99
AET	-1.00	0.83	0.93	0.99	0.79	0.99	1.00	1.00	1.00	1.00	1.00
MAP	-1.00	0.83	0.93	1.00	0.78	0.99	1.00	1.00	1.00	1.00	1.00
MAT	-0.99	0.81	0.92	0.99	0.78	1.00	1.00	0.99	1.00	1.00	1.00

Mt. Gaoligong-Shrub											
	Area	ISO	PS	PWQ	PDQ	Max	Min	Vapor	AET	MAP	MAT
Area	1.00	-0.74	-0.90	-0.99	-0.99	-0.79	-1.00	-1.00	-1.00	-1.00	-1.00
ISO	-0.74	1.00	0.96	0.69	0.65	0.87	0.76	0.77	0.74	0.74	0.70
PS	-0.90	0.96	1.00	0.87	0.84	0.91	0.92	0.93	0.90	0.90	0.88
PWQ	-0.99	0.69	0.87	1.00	1.00	0.72	0.99	0.99	0.99	1.00	1.00
PDQ	-0.99	0.65	0.84	1.00	1.00	0.72	0.99	0.98	0.99	0.99	1.00
Max	-0.79	0.87	0.91	0.72	0.72	1.00	0.81	0.83	0.79	0.78	0.77
Min	-1.00	0.76	0.92	0.99	0.99	0.81	1.00	1.00	1.00	1.00	1.00
Vapor	-1.00	0.77	0.93	0.99	0.98	0.83	1.00	1.00	1.00	1.00	0.99
AET	-1.00	0.74	0.90	0.99	0.99	0.79	1.00	1.00	1.00	1.00	1.00
MAP	-1.00	0.74	0.90	1.00	0.99	0.78	1.00	1.00	1.00	1.00	1.00
MAT	-1.00	0.70	0.88	1.00	1.00	0.77	1.00	0.99	1.00	1.00	1.00

Mt. Gaoligong-Herb											
	Area	ISO	PS	PWQ	PDQ	Max	Min	Vapor	AET	MAP	MAT
Area	1.00	-0.77	-0.92	-0.99	-0.79	-0.98	-1.00	-1.00	-1.00	-1.00	-0.99
ISO	-0.77	1.00	0.96	0.70	0.87	0.67	0.77	0.78	0.75	0.75	0.72
PS	-0.92	0.96	1.00	0.87	0.90	0.85	0.92	0.93	0.91	0.90	0.89
PWQ	-0.99	0.70	0.87	1.00	0.71	1.00	0.99	0.98	0.99	1.00	1.00
PDQ	-0.79	0.87	0.90	0.71	1.00	0.70	0.80	0.82	0.78	0.76	0.76
Max	-0.98	0.67	0.85	1.00	0.70	1.00	0.99	0.98	0.99	0.99	1.00
Min	-1.00	0.77	0.92	0.99	0.80	0.99	1.00	1.00	1.00	1.00	1.00
Vapor	-1.00	0.78	0.93	0.98	0.82	0.98	1.00	1.00	1.00	1.00	0.99
AET	-1.00	0.75	0.91	0.99	0.78	0.99	1.00	1.00	1.00	1.00	1.00
MAP	-1.00	0.75	0.90	1.00	0.76	0.99	1.00	1.00	1.00	1.00	1.00
MAT	-0.99	0.72	0.89	1.00	0.76	1.00	1.00	0.99	1.00	1.00	1.00

Mt. Gongga-Tree, Shrub, Herb											
	Area	ISO	PS	PWQ	PDQ	Max	Min	Vapor	AET	MAP	MAT
Area	1.00	0.83	1.00	-0.99	-0.22	0.98	-1.00	-1.00	-1.00	-1.00	-1.00
ISO	0.83	1.00	0.79	-0.89	-0.42	0.90	-0.84	-0.86	-0.81	-0.84	-0.82
PS	1.00	0.79	1.00	-0.98	-0.20	0.97	-1.00	-0.99	-1.00	-1.00	-1.00
PWQ	-0.99	-0.89	-0.98	1.00	0.33	-1.00	0.99	0.99	0.98	0.99	0.99
PDQ	-0.22	-0.42	-0.20	0.33	1.00	-0.34	0.22	0.25	0.20	0.22	0.21
Max	0.98	0.90	0.97	-1.00	-0.34	1.00	-0.99	-0.99	-0.98	-0.99	-0.98
Min	-1.00	-0.84	-1.00	0.99	0.22	-0.99	1.00	1.00	1.00	1.00	1.00
Vapor	-1.00	-0.86	-0.99	0.99	0.25	-0.99	1.00	1.00	1.00	1.00	1.00
AET	-1.00	-0.81	-1.00	0.98	0.20	-0.98	1.00	1.00	1.00	1.00	1.00
MAP	-1.00	-0.84	-1.00	0.99	0.22	-0.99	1.00	1.00	1.00	1.00	1.00
MAT	-1.00	-0.82	-1.00	0.99	0.21	-0.98	1.00	1.00	1.00	1.00	1.00

Mt. Haba-Tree, Shrub, Herb											
	Area	ISO	Map	Mat	Max	Min	Vapor	AET	PWQ	PDQ	PS
Area	1.00	-0.57	-0.76	-0.52	-0.52	-0.52	-0.58	-0.56	0.09	-0.70	0.56
ISO	-0.57	1.00	0.75	0.98	0.98	0.98	0.98	0.99	-0.79	0.85	-0.98
Map	-0.76	0.75	1.00	0.71	0.71	0.71	0.79	0.76	-0.25	0.93	-0.79
Mat	-0.52	0.98	0.71	1.00	1.00	1.00	0.99	1.00	-0.84	0.81	-0.98
Max	-0.52	0.98	0.71	1.00	1.00	1.00	0.99	1.00	-0.84	0.80	-0.97
Min	-0.52	0.98	0.71	1.00	1.00	1.00	0.99	1.00	-0.84	0.81	-0.98
Vapor	-0.58	0.98	0.79	0.99	0.99	0.99	1.00	1.00	-0.78	0.86	-0.99
AET	-0.56	0.99	0.76	1.00	1.00	1.00	1.00	1.00	-0.80	0.84	-0.98
PWQ	0.09	-0.79	-0.25	-0.84	-0.84	-0.84	-0.78	-0.80	1.00	-0.43	0.77
PDQ	-0.70	0.85	0.93	0.81	0.80	0.81	0.86	0.84	-0.43	1.00	-0.90
PS	0.56	-0.98	-0.79	-0.98	-0.97	-0.98	-0.99	-0.98	0.77	-0.90	1.00

Mt.Helan-Tree											
	Area	ISO	Map	Mat	Max	Min	Vapor	AET	PWQ	PDQ	PS
Area	1.00	0.98	-0.93	0.93	0.94	0.89	0.95	-0.98	-0.88	-0.36	-0.68
ISO	0.98	1.00	-0.97	0.97	0.98	0.94	0.98	-0.96	-0.94	-0.36	-0.77
Map	-0.93	-0.97	1.00	-1.00	-1.00	-0.99	-1.00	0.88	0.99	0.48	0.75
Mat	0.93	0.97	-1.00	1.00	1.00	0.99	1.00	-0.88	-0.99	-0.47	-0.76
Max	0.94	0.98	-1.00	1.00	1.00	0.99	1.00	-0.89	-0.99	-0.45	-0.76
Min	0.89	0.94	-0.99	0.99	0.99	1.00	0.99	-0.82	-0.98	-0.53	-0.72
Vapor	0.95	0.98	-1.00	1.00	1.00	0.99	1.00	-0.90	-0.98	-0.45	-0.76
AET	-0.98	-0.96	0.88	-0.88	-0.89	-0.82	-0.90	1.00	0.84	0.22	0.72
PWQ	-0.88	-0.94	0.99	-0.99	-0.99	-0.98	-0.98	0.84	1.00	0.42	0.82
PDQ	-0.36	-0.36	0.48	-0.47	-0.45	-0.53	-0.45	0.22	0.42	1.00	-0.14
PS	-0.68	-0.77	0.75	-0.76	-0.76	-0.72	-0.76	0.72	0.82	-0.14	1.00

Mt. Helan-Shrub											
	Area	ISO	Map	Mat	Max	Min	Vapor	AET	PWQ	PDQ	PS
Area	1.00	0.99	-0.94	0.95	0.95	0.91	0.96	-0.98	-0.90	-0.31	-0.70
ISO	0.99	1.00	-0.97	0.98	0.98	0.95	0.99	-0.97	-0.95	-0.30	-0.76
Map	-0.94	-0.97	1.00	-1.00	-1.00	-0.99	-1.00	0.89	0.99	0.42	0.75
Mat	0.95	0.98	-1.00	1.00	1.00	0.99	1.00	-0.90	-0.99	-0.41	-0.75
Max	0.95	0.98	-1.00	1.00	1.00	0.99	1.00	-0.91	-0.98	-0.39	-0.76
Min	0.91	0.95	-0.99	0.99	0.99	1.00	0.99	-0.84	-0.98	-0.47	-0.72
Vapor	0.96	0.99	-1.00	1.00	1.00	0.99	1.00	-0.92	-0.98	-0.39	-0.76
Wind	-0.87	-0.91	0.98	-0.98	-0.97	-0.99	-0.97	0.79	0.97	0.50	0.70
AET	-0.98	-0.97	0.89	-0.90	-0.91	-0.84	-0.92	1.00	0.86	0.18	0.73
PWQ	-0.90	-0.95	0.99	-0.99	-0.98	-0.98	-0.98	0.86	1.00	0.33	0.83
PDQ	-0.31	-0.30	0.42	-0.41	-0.39	-0.47	-0.39	0.18	0.33	1.00	-0.22

Mt. Helan-Herb											
	Area	ISO	Map	Mat	Max	Min	Vapor	AET	PWQ	PDQ	PS
Area	1.00	0.99	-0.94	0.94	0.95	0.91	0.96	-0.98	-0.90	-0.34	-0.70
ISO	0.99	1.00	-0.97	0.98	0.98	0.94	0.99	-0.97	-0.95	-0.34	-0.77
Map	-0.94	-0.97	1.00	-1.00	-1.00	-0.99	-1.00	0.89	0.99	0.46	0.75
Mat	0.94	0.98	-1.00	1.00	1.00	0.99	1.00	-0.89	-0.99	-0.45	-0.75
Max	0.95	0.98	-1.00	1.00	1.00	0.99	1.00	-0.90	-0.99	-0.43	-0.76
Min	0.91	0.94	-0.99	0.99	0.99	1.00	0.99	-0.84	-0.98	-0.51	-0.71
Vapor	0.96	0.99	-1.00	1.00	1.00	0.99	1.00	-0.91	-0.98	-0.42	-0.76
AET	-0.98	-0.97	0.89	-0.89	-0.90	-0.84	-0.91	1.00	0.86	0.20	0.73
PWQ	-0.90	-0.95	0.99	-0.99	-0.99	-0.98	-0.98	0.86	1.00	0.38	0.82
PDQ	-0.34	-0.34	0.46	-0.45	-0.43	-0.51	-0.42	0.20	0.38	1.00	-0.18
PS	-0.70	-0.77	0.75	-0.75	-0.76	-0.71	-0.76	0.73	0.82	-0.18	1.00

Mt. Himalaya (Indian)-Tree											
	Area	ISO	Map	Mat	Max	Min	Vapor	AET	PWQ	PDQ	PS
Area	1.00	-0.77	-0.26	-0.41	-0.42	-0.43	-0.27	-0.26	0.40	0.29	0.25
ISO	-0.77	1.00	0.24	0.41	0.42	0.44	0.23	0.24	-0.37	-0.09	-0.30
Map	-0.26	0.24	1.00	0.98	0.98	0.97	1.00	1.00	-0.96	-0.33	-0.74
Mat	-0.41	0.41	0.98	1.00	1.00	1.00	0.98	0.98	-0.99	-0.31	-0.78
Max	-0.42	0.42	0.98	1.00	1.00	1.00	0.98	0.98	-0.99	-0.30	-0.79
Min	-0.43	0.44	0.97	1.00	1.00	1.00	0.97	0.97	-0.99	-0.30	-0.78
Vapor	-0.27	0.23	1.00	0.98	0.98	0.97	1.00	1.00	-0.97	-0.34	-0.74
AET	-0.26	0.24	1.00	0.98	0.98	0.97	1.00	1.00	-0.96	-0.34	-0.74
PWQ	0.40	-0.37	-0.96	-0.99	-0.99	-0.99	-0.97	-0.96	1.00	0.20	0.86
PDQ	0.29	-0.09	-0.33	-0.31	-0.30	-0.30	-0.34	-0.34	0.20	1.00	-0.30
PS	0.25	-0.30	-0.74	-0.78	-0.79	-0.78	-0.74	-0.74	0.86	-0.30	1.00

Mt. Himalaya(Indian)-Shrub											
	Area	ISO	Map	Mat	Max	Min	Vapor	AET	PWQ	PDQ	PS
Area	1.00	-0.80	-0.48	-0.64	-0.63	-0.66	-0.50	-0.49	0.64	0.70	0.32
ISO	-0.80	1.00	0.40	0.56	0.57	0.59	0.40	0.39	-0.55	-0.42	-0.36
Map	-0.48	0.40	1.00	0.98	0.98	0.97	1.00	1.00	-0.96	-0.51	-0.75
Mat	-0.64	0.56	0.98	1.00	1.00	1.00	0.98	0.98	-0.99	-0.58	-0.76
Max	-0.63	0.57	0.98	1.00	1.00	1.00	0.98	0.98	-0.99	-0.56	-0.77
Min	-0.66	0.59	0.97	1.00	1.00	1.00	0.97	0.97	-0.99	-0.59	-0.75
Vapor	-0.50	0.40	1.00	0.98	0.98	0.97	1.00	1.00	-0.97	-0.53	-0.75
AET	-0.49	0.39	1.00	0.98	0.98	0.97	1.00	1.00	-0.96	-0.53	-0.75
PWQ	0.64	-0.55	-0.96	-0.99	-0.99	-0.99	-0.97	-0.96	1.00	0.53	0.82
PDQ	0.70	-0.42	-0.51	-0.58	-0.56	-0.59	-0.53	-0.53	0.53	1.00	-0.03
PS	0.32	-0.36	-0.75	-0.76	-0.77	-0.75	-0.75	-0.75	0.82	-0.03	1.00

Mt. Himalaya (Indian)-Herb											
	Area	ISO	Map	Mat	Max	Min	Vapor	AET	PWQ	PDQ	PS
Area	1.00	-0.80	-0.49	-0.65	-0.64	-0.67	-0.51	-0.50	0.65	0.81	0.27
ISO	-0.80	1.00	0.40	0.57	0.58	0.60	0.41	0.40	-0.53	-0.58	-0.25
Map	-0.49	0.40	1.00	0.98	0.98	0.97	1.00	1.00	-0.97	-0.50	-0.79
Mat	-0.65	0.57	0.98	1.00	1.00	1.00	0.98	0.98	-0.99	-0.61	-0.77
Max	-0.64	0.58	0.98	1.00	1.00	1.00	0.98	0.98	-0.99	-0.59	-0.78
Min	-0.67	0.60	0.97	1.00	1.00	1.00	0.97	0.97	-0.99	-0.63	-0.76
Vapor	-0.51	0.41	1.00	0.98	0.98	0.97	1.00	1.00	-0.97	-0.53	-0.79
AET	-0.50	0.40	1.00	0.98	0.98	0.97	1.00	1.00	-0.97	-0.52	-0.79
PWQ	0.65	-0.53	-0.97	-0.99	-0.99	-0.99	-0.97	-0.97	1.00	0.58	0.82
PDQ	0.81	-0.58	-0.50	-0.61	-0.59	-0.63	-0.53	-0.52	0.58	1.00	0.04
PS	0.27	-0.25	-0.79	-0.77	-0.78	-0.76	-0.79	-0.79	0.82	0.04	1.00

Mt. Himalaya (Nepal)-Tree											
	Area	ISO	Map	Mat	Max	Min	Vapor	AET	PWQ	PDQ	PS
Area	1.00	-0.12	-0.10	-0.09	0.01	-0.18	-0.15	-0.06	0.05	0.20	-0.01
ISO	-0.12	1.00	0.99	0.99	0.98	0.99	0.99	0.99	-0.99	-0.99	-0.96
Map	-0.10	0.99	1.00	1.00	0.99	0.99	1.00	1.00	-1.00	-0.99	-0.95
Mat	-0.09	0.99	1.00	1.00	0.99	1.00	1.00	1.00	-1.00	-0.99	-0.96
Max	0.01	0.98	0.99	0.99	1.00	0.98	0.99	1.00	-1.00	-0.97	-0.96
Min	-0.18	0.99	0.99	1.00	0.98	1.00	1.00	0.99	-0.99	-1.00	-0.95
Vapor	-0.15	0.99	1.00	1.00	0.99	1.00	1.00	1.00	-0.99	-0.99	-0.95
AET	-0.06	0.99	1.00	1.00	1.00	0.99	1.00	1.00	-1.00	-0.99	-0.95
PWQ	0.05	-0.99	-1.00	-1.00	-1.00	-0.99	-0.99	-1.00	1.00	0.99	0.95
PDQ	0.20	-0.99	-0.99	-0.99	-0.97	-1.00	-0.99	-0.99	0.99	1.00	0.93
PS	-0.01	-0.96	-0.95	-0.96	-0.96	-0.95	-0.95	-0.95	0.95	0.93	1.00

Mt. Himalaya (Nepal)-Shrub											
	Area	ISO	Map	Mat	Max	Min	Vapor	AET	PWQ	PDQ	PS
Area	1.00	-0.12	-0.09	-0.09	0.01	-0.18	-0.14	-0.06	0.06	0.21	-0.01
ISO	-0.12	1.00	0.99	0.99	0.98	0.99	0.99	0.99	-0.99	-0.99	-0.97
Map	-0.09	0.99	1.00	1.00	0.99	0.99	1.00	1.00	-1.00	-0.98	-0.95
Mat	-0.09	0.99	1.00	1.00	0.99	1.00	1.00	1.00	-1.00	-0.99	-0.96
Max	0.01	0.98	0.99	0.99	1.00	0.98	0.99	1.00	-1.00	-0.97	-0.96
Min	-0.18	0.99	0.99	1.00	0.98	1.00	1.00	0.99	-0.99	-0.99	-0.95
Vapor	-0.14	0.99	1.00	1.00	0.99	1.00	1.00	1.00	-0.99	-0.99	-0.95
AET	-0.06	0.99	1.00	1.00	1.00	0.99	1.00	1.00	-1.00	-0.98	-0.95
PWQ	0.06	-0.99	-1.00	-1.00	-1.00	-0.99	-0.99	-1.00	1.00	0.99	0.95
PDQ	0.21	-0.99	-0.98	-0.99	-0.97	-0.99	-0.99	-0.98	0.99	1.00	0.93
PS	-0.01	-0.97	-0.95	-0.96	-0.96	-0.95	-0.95	-0.95	0.95	0.93	1.00

Mt. Himalaya (Nepal)-Herb											
	Area	ISO	Map	Mat	Max	Min	Vapor	AET	PWQ	PDQ	PS
Area	1.00	-0.26	-0.26	-0.26	-0.14	-0.35	-0.31	-0.22	0.21	0.38	0.07
ISO	-0.26	1.00	0.99	0.99	0.98	0.99	0.99	0.99	-0.98	-0.98	-0.95
Map	-0.26	0.99	1.00	1.00	0.99	0.99	1.00	1.00	-1.00	-0.98	-0.95
Mat	-0.26	0.99	1.00	1.00	0.99	1.00	1.00	1.00	-1.00	-0.99	-0.95
Max	-0.14	0.98	0.99	0.99	1.00	0.98	0.98	1.00	-1.00	-0.96	-0.97
Min	-0.35	0.99	0.99	1.00	0.98	1.00	1.00	0.99	-0.98	-1.00	-0.93
Vapor	-0.31	0.99	1.00	1.00	0.98	1.00	1.00	1.00	-0.99	-0.99	-0.94
AET	-0.22	0.99	1.00	1.00	1.00	0.99	1.00	1.00	-1.00	-0.98	-0.96
PWQ	0.21	-0.98	-1.00	-1.00	-1.00	-0.98	-0.99	-1.00	1.00	0.98	0.96
PDQ	0.38	-0.98	-0.98	-0.99	-0.96	-1.00	-0.99	-0.98	0.98	1.00	0.91
PS	0.07	-0.95	-0.95	-0.95	-0.97	-0.93	-0.94	-0.96	0.96	0.91	1.00

Mt. Jiuding-Tree											
	Area	ISO	Map	Mat	Max	Min	Vapor	AET	PWQ	PDQ	PS
Area	1.00	0.69	-0.47	-0.73	-0.73	-0.75	-0.75	-0.68	-0.20	-0.52	-0.04
ISO	0.69	1.00	-0.96	-1.00	-1.00	-0.99	-1.00	-1.00	-0.84	-0.80	-0.73
Map	-0.47	-0.96	1.00	0.94	0.95	0.93	0.93	0.97	0.95	0.78	0.88
Mat	-0.73	-1.00	0.94	1.00	1.00	1.00	1.00	1.00	0.81	0.78	0.70
Max	-0.73	-1.00	0.95	1.00	1.00	1.00	1.00	1.00	0.81	0.78	0.70
Min	-0.75	-0.99	0.93	1.00	1.00	1.00	1.00	0.99	0.80	0.77	0.68
Vapor	-0.75	-1.00	0.93	1.00	1.00	1.00	1.00	0.99	0.78	0.79	0.66
AET	-0.68	-1.00	0.97	1.00	1.00	0.99	0.99	1.00	0.85	0.78	0.75
PWQ	-0.20	-0.84	0.95	0.81	0.81	0.80	0.78	0.85	1.00	0.64	0.98
PDQ	-0.52	-0.80	0.78	0.78	0.78	0.77	0.79	0.78	0.64	1.00	0.48
PS	-0.04	-0.73	0.88	0.70	0.70	0.68	0.66	0.75	0.98	0.48	1.00

Mt. Jiuding-Shrub, Herb											
	Area	ISO	Map	Mat	Max	Min	Vapor	AET	PWQ	PDQ	PS
Area	1.00	0.76	-0.53	-0.77	-0.76	-0.78	-0.79	-0.74	-0.22	-0.41	-0.10
ISO	0.76	1.00	-0.91	-0.97	-0.97	-0.97	-0.99	-0.98	-0.75	-0.62	-0.67
Map	-0.53	-0.91	1.00	0.82	0.82	0.81	0.84	0.86	0.94	0.73	0.87
Mat	-0.77	-0.97	0.82	1.00	1.00	1.00	0.99	1.00	0.64	0.48	0.58
Max	-0.76	-0.97	0.82	1.00	1.00	1.00	1.00	1.00	0.65	0.49	0.58
Min	-0.78	-0.97	0.81	1.00	1.00	1.00	0.99	0.99	0.63	0.47	0.56
Vapor	-0.79	-0.99	0.84	0.99	1.00	0.99	1.00	0.99	0.66	0.54	0.58
AET	-0.74	-0.98	0.86	1.00	1.00	0.99	0.99	1.00	0.70	0.51	0.64
PWQ	-0.22	-0.75	0.94	0.64	0.65	0.63	0.66	0.70	1.00	0.62	0.98
PDQ	-0.41	-0.62	0.73	0.48	0.49	0.47	0.54	0.51	0.62	1.00	0.46
PS	-0.10	-0.67	0.87	0.58	0.58	0.56	0.58	0.64	0.98	0.46	1.00

Mt. Miandam-Tree, Shrub, Herb											
	Area	ISO	Map	Mat	Max	Min	Vapor	AET	PWQ	PDQ	PS
Area	1.00	0.93	-0.11	0.91	0.89	0.94	0.86	0.91	-0.49	0.76	0.26
ISO	0.93	1.00	-0.35	0.99	0.99	1.00	0.98	0.99	-0.44	0.93	0.48
Map	-0.11	-0.35	1.00	-0.36	-0.38	-0.33	-0.40	-0.35	0.15	-0.48	-0.40
Mat	0.91	0.99	-0.36	1.00	1.00	1.00	0.99	1.00	-0.38	0.96	0.55
Max	0.89	0.99	-0.38	1.00	1.00	0.99	1.00	1.00	-0.36	0.97	0.58
Min	0.94	1.00	-0.33	1.00	0.99	1.00	0.98	1.00	-0.41	0.94	0.51
Vapor	0.86	0.98	-0.40	0.99	1.00	0.98	1.00	0.99	-0.32	0.98	0.63
AET	0.91	0.99	-0.35	1.00	1.00	1.00	0.99	1.00	-0.38	0.96	0.54
PWQ	-0.49	-0.44	0.15	-0.38	-0.36	-0.41	-0.32	-0.38	1.00	-0.21	0.21
PDQ	0.76	0.93	-0.48	0.96	0.97	0.94	0.98	0.96	-0.21	1.00	0.69
PS	0.26	0.48	-0.40	0.55	0.58	0.51	0.63	0.54	0.21	0.69	1.00

Mt. Naeba-Tree											
	Area	ISO	Map	Mat	Max	Min	Vapor	AET	PWQ	PDQ	PS
Area	1.00	0.83	-0.95	0.98	0.98	0.98	0.99	0.99	-0.80	0.65	-0.98
ISO	0.83	1.00	-0.94	0.89	0.89	0.88	0.85	0.87	-0.97	0.16	-0.72
Map	-0.95	-0.94	1.00	-0.99	-0.99	-0.98	-0.97	-0.98	0.94	-0.42	0.88
Mat	0.98	0.89	-0.99	1.00	1.00	1.00	1.00	1.00	-0.87	0.55	-0.94
Max	0.98	0.89	-0.99	1.00	1.00	1.00	1.00	1.00	-0.88	0.55	-0.94
Min	0.98	0.88	-0.98	1.00	1.00	1.00	1.00	1.00	-0.87	0.56	-0.94
Vapor	0.99	0.85	-0.97	1.00	1.00	1.00	1.00	1.00	-0.83	0.62	-0.96
AET	0.99	0.87	-0.98	1.00	1.00	1.00	1.00	1.00	-0.85	0.59	-0.95
PWQ	-0.80	-0.97	0.94	-0.87	-0.88	-0.87	-0.83	-0.85	1.00	-0.10	0.68
PDQ	0.65	0.16	-0.42	0.55	0.55	0.56	0.62	0.59	-0.10	1.00	-0.79
PS	-0.98	-0.72	0.88	-0.94	-0.94	-0.94	-0.96	-0.95	0.68	-0.79	1.00

Mt. Naeba-Herb											
	Area	ISO	Map	Mat	Max	Min	Vapor	AET	PWQ	PDQ	PS
Area	1.00	0.87	-0.95	0.98	0.98	0.98	0.99	0.99	-0.83	0.64	-0.98
ISO	0.87	1.00	-0.95	0.92	0.92	0.92	0.89	0.91	-0.96	0.22	-0.77
Map	-0.95	-0.95	1.00	-0.99	-0.99	-0.99	-0.98	-0.98	0.95	-0.42	0.89
Mat	0.98	0.92	-0.99	1.00	1.00	1.00	1.00	1.00	-0.90	0.54	-0.94
Max	0.98	0.92	-0.99	1.00	1.00	1.00	1.00	1.00	-0.90	0.54	-0.94
Min	0.98	0.92	-0.99	1.00	1.00	1.00	1.00	1.00	-0.90	0.55	-0.95
Vapor	0.99	0.89	-0.98	1.00	1.00	1.00	1.00	1.00	-0.86	0.60	-0.96
AET	0.99	0.91	-0.98	1.00	1.00	1.00	1.00	1.00	-0.88	0.57	-0.95
PWQ	-0.83	-0.96	0.95	-0.90	-0.90	-0.90	-0.86	-0.88	1.00	-0.15	0.73
PDQ	0.64	0.22	-0.42	0.54	0.54	0.55	0.60	0.57	-0.15	1.00	-0.77
PS	-0.98	-0.77	0.89	-0.94	-0.94	-0.95	-0.96	-0.95	0.73	-0.77	1.00

Mt. Naeba-Fern											
	Area	ISO	Map	Mat	Max	Min	Vapor	AET	PWQ	PDQ	PS
Area	1.00	0.72	-0.95	0.98	0.98	0.99	0.99	0.99	-0.71	0.70	-0.97
ISO	0.72	1.00	-0.88	0.77	0.77	0.76	0.71	0.74	-0.94	0.08	-0.54
Map	-0.95	-0.88	1.00	-0.98	-0.98	-0.97	-0.95	-0.96	0.88	-0.49	0.85
Mat	0.98	0.77	-0.98	1.00	1.00	1.00	1.00	1.00	-0.77	0.66	-0.94
Max	0.98	0.77	-0.98	1.00	1.00	1.00	0.99	1.00	-0.78	0.66	-0.94
Min	0.99	0.76	-0.97	1.00	1.00	1.00	1.00	1.00	-0.76	0.68	-0.95
Vapor	0.99	0.71	-0.95	1.00	0.99	1.00	1.00	1.00	-0.71	0.73	-0.97
AET	0.99	0.74	-0.96	1.00	1.00	1.00	1.00	1.00	-0.74	0.70	-0.96
PWQ	-0.71	-0.94	0.88	-0.77	-0.78	-0.76	-0.71	-0.74	1.00	-0.05	0.53
PDQ	0.70	0.08	-0.49	0.66	0.66	0.68	0.73	0.70	-0.05	1.00	-0.86
PS	-0.97	-0.54	0.85	-0.94	-0.94	-0.95	-0.97	-0.96	0.53	-0.86	1.00

Mt. Niubeiliang-Tree, Shrub											
	Area	ISO	PS	PWQ	PDQ	Max	Min	Vapor	AET	MAP	MAT
Area	1.00	-0.96	-0.97	0.84	0.70	0.17	-1.00	-1.00	-1.00	-1.00	1.00
ISO	-0.96	1.00	1.00	-0.86	-0.74	-0.14	0.97	0.95	0.97	0.98	-0.94
PS	-0.97	1.00	1.00	-0.84	-0.75	-0.18	0.98	0.97	0.98	0.98	-0.95
PWQ	0.84	-0.86	-0.84	1.00	0.36	-0.36	-0.84	-0.83	-0.84	-0.84	0.84
PDQ	0.70	-0.74	-0.75	0.36	1.00	0.71	-0.72	-0.71	-0.72	-0.73	0.68
Max	0.17	-0.14	-0.18	-0.36	0.71	1.00	-0.18	-0.18	-0.17	-0.18	0.15
Min	-1.00	0.97	0.98	-0.84	-0.72	-0.18	1.00	1.00	1.00	1.00	-0.99
Vapor	-1.00	0.95	0.97	-0.83	-0.71	-0.18	1.00	1.00	1.00	1.00	-1.00
AET	-1.00	0.97	0.98	-0.84	-0.72	-0.17	1.00	1.00	1.00	1.00	-0.99
MAP	-1.00	0.98	0.98	-0.84	-0.73	-0.18	1.00	1.00	1.00	1.00	-0.99
MAT	1.00	-0.94	-0.95	0.84	0.68	0.15	-0.99	-1.00	-0.99	-0.99	1.00

Mt. Niubeiliang-Herb											
	Area	ISO	PS	PWQ	PDQ	Max	Min	Vapor	AET	MAP	MAT
Area	1.00	1.00	-0.85	-0.77	-0.17	0.96	0.95	0.97	0.97	-0.94	0.96
ISO	1.00	1.00	-0.84	-0.78	-0.21	0.97	0.96	0.98	0.98	-0.95	0.97
PS	-0.85	-0.84	1.00	0.40	-0.33	-0.84	-0.83	-0.85	-0.85	0.85	-0.84
PWQ	-0.77	-0.78	0.40	1.00	0.71	-0.76	-0.75	-0.75	-0.76	0.73	-0.75
PDQ	-0.17	-0.21	-0.33	0.71	1.00	-0.20	-0.21	-0.19	-0.20	0.18	-0.20
Max	0.96	0.97	-0.84	-0.76	-0.20	1.00	1.00	1.00	1.00	-0.99	1.00
Min	0.95	0.96	-0.83	-0.75	-0.21	1.00	1.00	1.00	1.00	-1.00	1.00
Vapor	0.97	0.98	-0.85	-0.75	-0.19	1.00	1.00	1.00	1.00	-0.99	1.00
AET	0.97	0.98	-0.85	-0.76	-0.20	1.00	1.00	1.00	1.00	-0.99	1.00
MAP	-0.94	-0.95	0.85	0.73	0.18	-0.99	-1.00	-0.99	-0.99	1.00	-0.99
MAT	0.96	0.97	-0.84	-0.75	-0.20	1.00	1.00	1.00	1.00	-0.99	1.00

Mt. Popocatepetl-Tree

	Area	ISO	Map	Mat	Max	Min	Vapor	AET	PWQ	PDQ	PS
Area	1	-0.89	-0.83	0.85	0.85	0.86	0.87	0.94	-0.9	-0.94	-0.8
ISO	-0.89	1	0.76	-0.77	-0.76	-0.76	-0.82	-0.89	0.89	0.95	0.86
Map	-0.83	0.76	1	-1	-1	-0.99	-0.99	-0.94	0.97	0.91	0.81
Mat	0.85	-0.77	-1	1	1	1	1	0.96	-0.97	-0.92	-0.82
Max	0.85	-0.76	-1	1	1	1	1	0.96	-0.97	-0.91	-0.81
Min	0.86	-0.76	-0.99	1	1	1	0.99	0.96	-0.96	-0.92	-0.8
Vapor	0.87	-0.82	-0.99	1	1	0.99	1	0.97	-0.99	-0.94	-0.85
AET	0.94	-0.89	-0.94	0.96	0.96	0.96	0.97	1	-0.98	-0.97	-0.89
PWQ	-0.9	0.89	0.97	-0.97	-0.97	-0.96	-0.99	-0.98	1	0.98	0.91
PDQ	-0.94	0.95	0.91	-0.92	-0.91	-0.92	-0.94	-0.97	0.98	1	0.87
PS	-0.8	0.86	0.81	-0.82	-0.81	-0.8	-0.85	-0.89	0.91	0.87	1

Mt. Popocatepetl-Shrub

	Area	ISO	Map	Mat	Max	Min	Vapor	AET	PWQ	PDQ	PS
Area	1.00	-0.89	-0.84	0.86	0.86	0.87	0.87	0.94	-0.90	-0.94	-0.81
ISO	-0.89	1.00	0.76	-0.78	-0.77	-0.77	-0.82	-0.89	0.89	0.95	0.86
Map	-0.84	0.76	1.00	-1.00	-1.00	-1.00	-0.99	-0.94	0.97	0.91	0.81
Mat	0.86	-0.78	-1.00	1.00	1.00	1.00	1.00	0.96	-0.97	-0.92	-0.82
Max	0.86	-0.77	-1.00	1.00	1.00	1.00	1.00	0.96	-0.97	-0.92	-0.82
Min	0.87	-0.77	-1.00	1.00	1.00	1.00	0.99	0.96	-0.97	-0.92	-0.81
Vapor	0.87	-0.82	-0.99	1.00	1.00	0.99	1.00	0.97	-0.99	-0.95	-0.85
AET	0.94	-0.89	-0.94	0.96	0.96	0.96	0.97	1.00	-0.98	-0.97	-0.89
PWQ	-0.90	0.89	0.97	-0.97	-0.97	-0.97	-0.99	-0.98	1.00	0.98	0.91
PDQ	-0.94	0.95	0.91	-0.92	-0.92	-0.92	-0.95	-0.97	0.98	1.00	0.87
PS	-0.81	0.86	0.81	-0.82	-0.82	-0.81	-0.85	-0.89	0.91	0.87	1.00

Mt. Popocatepetl-Herb											
	Area	ISO	Map	Mat	Max	Min	Vapor	AET	PWQ	PDQ	PS
Area	1.00	-0.89	-0.83	0.85	0.85	0.86	0.87	0.94	-0.87	-0.93	-0.83
ISO	-0.89	1.00	0.76	-0.77	-0.76	-0.76	-0.82	-0.89	0.84	0.93	0.78
Map	-0.83	0.76	1.00	-1.00	-1.00	-0.99	-0.99	-0.94	0.99	0.94	0.85
Mat	0.85	-0.77	-1.00	1.00	1.00	1.00	1.00	0.96	-0.99	-0.95	-0.86
Max	0.85	-0.76	-1.00	1.00	1.00	1.00	1.00	0.96	-0.99	-0.95	-0.86
Min	0.86	-0.76	-0.99	1.00	1.00	1.00	0.99	0.96	-0.98	-0.95	-0.85
Vapor	0.87	-0.82	-0.99	1.00	1.00	0.99	1.00	0.97	-1.00	-0.97	-0.87
AET	0.94	-0.89	-0.94	0.96	0.96	0.96	0.97	1.00	-0.98	-0.99	-0.90
PWQ	-0.87	0.84	0.99	-0.99	-0.99	-0.98	-1.00	-0.98	1.00	0.97	0.90
PDQ	-0.93	0.93	0.94	-0.95	-0.95	-0.95	-0.97	-0.99	0.97	1.00	0.86
PS	-0.83	0.78	0.85	-0.86	-0.86	-0.85	-0.87	-0.90	0.90	0.86	1.00

Mt. Sela-Tree											
	Area	ISO	PS	PWQ	PDQ	Max	Min	Vapor	AET	MAP	MAT
Area	1.00	-0.76	-0.89	-0.47	0.93	-0.90	-0.91	-0.89	-0.89	-0.76	-0.90
ISO	-0.76	1.00	0.94	0.43	-0.89	0.92	0.91	0.90	0.93	0.91	0.92
PS	-0.89	0.94	1.00	0.54	-0.98	0.98	0.98	0.98	0.99	0.95	0.98
PWQ	-0.47	0.43	0.54	1.00	-0.60	0.65	0.66	0.68	0.65	0.69	0.65
PDQ	0.93	-0.89	-0.98	-0.60	1.00	-0.98	-0.99	-0.98	-0.98	-0.91	-0.99
Max	-0.90	0.92	0.98	0.65	-0.98	1.00	1.00	1.00	1.00	0.96	1.00
Min	-0.91	0.91	0.98	0.66	-0.99	1.00	1.00	1.00	1.00	0.95	1.00
Vapor	-0.89	0.90	0.98	0.68	-0.98	1.00	1.00	1.00	1.00	0.96	1.00
AET	-0.89	0.93	0.99	0.65	-0.98	1.00	1.00	1.00	1.00	0.96	1.00
MAP	-0.76	0.91	0.95	0.69	-0.91	0.96	0.95	0.96	0.96	1.00	0.95
MAT	-0.90	0.92	0.98	0.65	-0.99	1.00	1.00	1.00	1.00	0.95	1.00

Mt. Sela-Shrub, Herb											
	Area	ISO	PS	PWQ	PDQ	Max	Min	Vapor	AET	MAP	MAT
Area	1.00	-0.71	-0.85	-0.33	0.85	-0.83	-0.84	-0.82	-0.82	-0.69	-0.83
ISO	-0.71	1.00	0.95	0.37	-0.91	0.93	0.92	0.92	0.94	0.90	0.93
PS	-0.85	0.95	1.00	0.49	-0.98	0.99	0.99	0.99	0.99	0.92	0.99
PWQ	-0.33	0.37	0.49	1.00	-0.44	0.52	0.52	0.56	0.53	0.70	0.52
PDQ	0.85	-0.91	-0.98	-0.44	1.00	-0.99	-0.99	-0.98	-0.98	-0.86	-0.99
Max	-0.83	0.93	0.99	0.52	-0.99	1.00	1.00	1.00	1.00	0.92	1.00
Min	-0.84	0.92	0.99	0.52	-0.99	1.00	1.00	1.00	1.00	0.91	1.00
Vapor	-0.82	0.92	0.99	0.56	-0.98	1.00	1.00	1.00	1.00	0.93	1.00
AET	-0.82	0.94	0.99	0.53	-0.98	1.00	1.00	1.00	1.00	0.93	1.00
MAP	-0.69	0.90	0.92	0.70	-0.86	0.92	0.91	0.93	0.93	1.00	0.92
MAT	-0.83	0.93	0.99	0.52	-0.99	1.00	1.00	1.00	1.00	0.92	1.00

Mt. Tai-Tree											
	Area	ISO	Map	Mat	Max	Min	Vapor	AET	PWQ	PDQ	PS
Area	1.00	0.39	-0.91	0.93	0.94	0.89	0.93	0.92	-0.88	0.26	0.59
ISO	0.39	1.00	0.00	0.06	0.10	-0.04	0.08	0.05	0.01	0.53	-0.19
Map	-0.91	0.00	1.00	-1.00	-0.99	-1.00	-1.00	-1.00	0.95	-0.16	-0.64
Mat	0.93	0.06	-1.00	1.00	1.00	0.99	1.00	1.00	-0.94	0.21	0.62
Max	0.94	0.10	-0.99	1.00	1.00	0.99	1.00	1.00	-0.94	0.23	0.61
Min	0.89	-0.04	-1.00	0.99	0.99	1.00	0.99	1.00	-0.95	0.15	0.65
Vapor	0.93	0.08	-1.00	1.00	1.00	0.99	1.00	1.00	-0.94	0.22	0.62
AET	0.92	0.05	-1.00	1.00	1.00	1.00	1.00	1.00	-0.95	0.20	0.63
PWQ	-0.88	0.01	0.95	-0.94	-0.94	-0.95	-0.94	-0.95	1.00	0.06	-0.83
PDQ	0.26	0.53	-0.16	0.21	0.23	0.15	0.22	0.20	0.06	1.00	-0.57
PS	0.59	-0.19	-0.64	0.62	0.61	0.65	0.62	0.63	-0.83	-0.57	1.00

Mt. Tai-Shrub, Herb											
	Area	ISO	Map	Mat	Max	Min	Vapor	AET	PWQ	PDQ	PS
Area	1.00	0.38	-0.91	0.93	0.94	0.89	0.93	0.93	-0.89	0.22	0.60
ISO	0.38	1.00	0.00	0.06	0.09	-0.04	0.07	0.05	0.02	0.53	-0.20
Map	-0.91	0.00	1.00	-1.00	-0.99	-1.00	-1.00	-1.00	0.95	-0.12	-0.65
Mat	0.93	0.06	-1.00	1.00	1.00	0.99	1.00	1.00	-0.94	0.17	0.63
Max	0.94	0.09	-0.99	1.00	1.00	0.99	1.00	1.00	-0.94	0.19	0.63
Min	0.89	-0.04	-1.00	0.99	0.99	1.00	0.99	1.00	-0.95	0.11	0.66
Vapor	0.93	0.07	-1.00	1.00	1.00	0.99	1.00	1.00	-0.94	0.18	0.63
AET	0.93	0.05	-1.00	1.00	1.00	1.00	1.00	1.00	-0.95	0.16	0.64
PWQ	-0.89	0.02	0.95	-0.94	-0.94	-0.95	-0.94	-0.95	1.00	0.10	-0.83
PDQ	0.22	0.53	-0.12	0.17	0.19	0.11	0.18	0.16	0.10	1.00	-0.60
PS	0.60	-0.20	-0.65	0.63	0.63	0.66	0.63	0.64	-0.83	-0.60	1.00

Mt. Taibai-Tree											
	Area	ISO	PS	PWQ	PDQ	Max	Min	Vapor	AET	MAP	MAT
Area	1.00	-0.73	-0.51	0.04	0.25	-0.39	-0.34	-0.41	-0.42	0.30	-0.37
ISO	-0.73	1.00	0.90	-0.64	-0.76	0.72	0.70	0.73	0.73	-0.64	0.71
PS	-0.51	0.90	1.00	-0.82	-0.76	0.57	0.59	0.58	0.58	-0.51	0.58
PWQ	0.04	-0.64	-0.82	1.00	0.90	-0.68	-0.72	-0.67	-0.67	0.68	-0.70
PDQ	0.25	-0.76	-0.76	0.90	1.00	-0.92	-0.94	-0.92	-0.92	0.92	-0.93
Max	-0.39	0.72	0.57	-0.68	-0.92	1.00	1.00	1.00	1.00	-0.99	1.00
Min	-0.34	0.70	0.59	-0.72	-0.94	1.00	1.00	1.00	1.00	-1.00	1.00
Vapor	-0.41	0.73	0.58	-0.67	-0.92	1.00	1.00	1.00	1.00	-0.99	1.00
AET	-0.42	0.73	0.58	-0.67	-0.92	1.00	1.00	1.00	1.00	-0.99	1.00
MAP	0.30	-0.64	-0.51	0.68	0.92	-0.99	-1.00	-0.99	-0.99	1.00	-1.00
MAT	-0.37	0.71	0.58	-0.70	-0.93	1.00	1.00	1.00	1.00	-1.00	1.00

Mt. Taibai-Shrub, Herb											
	Area	ISO	PS	PWQ	PDQ	Max	Min	Vapor	AET	MAP	MAT
Area	1.00	-0.35	0.38	-0.47	-0.62	0.37	0.42	0.35	0.34	-0.10	0.40
ISO	-0.35	1.00	0.64	-0.59	-0.44	0.60	0.57	0.61	0.61	-0.51	0.58
PS	0.38	0.64	1.00	-0.88	-0.90	0.78	0.80	0.78	0.78	-0.39	0.79
PWQ	-0.47	-0.59	-0.88	1.00	0.87	-0.84	-0.86	-0.83	-0.83	0.58	-0.85
PDQ	-0.62	-0.44	-0.90	0.87	1.00	-0.91	-0.93	-0.90	-0.90	0.50	-0.92
Max	0.37	0.60	0.78	-0.84	-0.91	1.00	1.00	1.00	1.00	-0.73	1.00
Min	0.42	0.57	0.80	-0.86	-0.93	1.00	1.00	1.00	1.00	-0.72	1.00
Vapor	0.35	0.61	0.78	-0.83	-0.90	1.00	1.00	1.00	1.00	-0.73	1.00
AET	0.34	0.61	0.78	-0.83	-0.90	1.00	1.00	1.00	1.00	-0.73	1.00
MAP	-0.10	-0.51	-0.39	0.58	0.50	-0.73	-0.72	-0.73	-0.73	1.00	-0.72
MAT	0.40	0.58	0.79	-0.85	-0.92	1.00	1.00	1.00	1.00	-0.72	1.00

Mt. Xiaowutai-Tree											
	Area	ISO	PS	PWQ	PDQ	Max	Min	Vapor	AET	MAP	MAT
Area	1.00	0.84	0.99	-0.56	-0.90	0.92	0.94	0.93	0.93	-0.93	0.93
ISO	0.84	1.00	0.88	-0.81	-0.94	0.93	0.93	0.93	0.94	-0.92	0.94
PS	0.99	0.88	1.00	-0.62	-0.93	0.95	0.96	0.95	0.95	-0.95	0.95
PWQ	-0.56	-0.81	-0.62	1.00	0.83	-0.83	-0.79	-0.82	-0.82	0.82	-0.82
PDQ	-0.90	-0.94	-0.93	0.83	1.00	-0.97	-0.96	-0.97	-0.97	0.96	-0.97
Max	0.92	0.93	0.95	-0.83	-0.97	1.00	0.99	1.00	1.00	-1.00	1.00
Min	0.94	0.93	0.96	-0.79	-0.96	0.99	1.00	0.99	1.00	-0.99	1.00
Vapor	0.93	0.93	0.95	-0.82	-0.97	1.00	0.99	1.00	1.00	-1.00	1.00
AET	0.93	0.94	0.95	-0.82	-0.97	1.00	1.00	1.00	1.00	-1.00	1.00
MAP	-0.93	-0.92	-0.95	0.82	0.96	-1.00	-0.99	-1.00	-1.00	1.00	-1.00
MAT	0.93	0.94	0.95	-0.82	-0.97	1.00	1.00	1.00	1.00	-1.00	1.00

Mt. Xiaowutai-Shrub, Herb											
	Area	ISO	PS	PWQ	PDQ	Max	Min	Vapor	AET	MAP	MAT
Area	1.00	0.88	0.99	-0.45	-0.88	0.94	0.96	0.95	0.95	-0.93	0.95
ISO	0.88	1.00	0.90	-0.67	-0.93	0.93	0.93	0.93	0.93	-0.92	0.93
PS	0.99	0.90	1.00	-0.48	-0.90	0.96	0.97	0.97	0.96	-0.95	0.96
PWQ	-0.45	-0.67	-0.48	1.00	0.78	-0.71	-0.64	-0.68	-0.69	0.72	-0.69
PDQ	-0.88	-0.93	-0.90	0.78	1.00	-0.96	-0.93	-0.94	-0.95	0.96	-0.95
Max	0.94	0.93	0.96	-0.71	-0.96	1.00	0.99	1.00	1.00	-1.00	1.00
Min	0.96	0.93	0.97	-0.64	-0.93	0.99	1.00	1.00	1.00	-0.98	1.00
Vapor	0.95	0.93	0.97	-0.68	-0.94	1.00	1.00	1.00	1.00	-0.99	1.00
AET	0.95	0.93	0.96	-0.69	-0.95	1.00	1.00	1.00	1.00	-0.99	1.00
MAP	-0.93	-0.92	-0.95	0.72	0.96	-1.00	-0.98	-0.99	-0.99	1.00	-0.99
MAT	0.95	0.93	0.96	-0.69	-0.95	1.00	1.00	1.00	1.00	-0.99	1.00

Mt. Yulong-Tree, Shrub, Herb											
	Area	ISO	PS	PWQ	PDQ	Max	Min	Vapor	AET	MAP	MAT
Area	1.00	0.99	-0.17	-0.60	0.64	0.99	1.00	1.00	1.00	0.99	1.00
ISO	0.99	1.00	-0.17	-0.50	0.70	0.97	0.98	0.99	0.99	1.00	0.98
PS	-0.17	-0.17	1.00	0.12	-0.69	-0.20	-0.18	-0.15	-0.15	-0.20	-0.19
PWQ	-0.60	-0.50	0.12	1.00	-0.04	-0.65	-0.64	-0.60	-0.61	-0.50	-0.64
PDQ	0.64	0.70	-0.69	-0.04	1.00	0.61	0.62	0.63	0.62	0.72	0.62
Max	0.99	0.97	-0.20	-0.65	0.61	1.00	1.00	0.99	1.00	0.97	1.00
Min	1.00	0.98	-0.18	-0.64	0.62	1.00	1.00	1.00	1.00	0.98	1.00
Vapor	1.00	0.99	-0.15	-0.60	0.63	0.99	1.00	1.00	1.00	0.99	1.00
AET	1.00	0.99	-0.15	-0.61	0.62	1.00	1.00	1.00	1.00	0.98	1.00
MAP	0.99	1.00	-0.20	-0.50	0.72	0.97	0.98	0.99	0.98	1.00	0.98
MAT	1.00	0.98	-0.19	-0.64	0.62	1.00	1.00	1.00	1.00	0.98	1.00

Mt. Baima-Tree, Shrub, Herb											
	Area	ISO	Map	Mat	Max	Min	Vapor	AET	PWQ	PDQ	PS
Area	1	-0.96	-0.99	-0.91	-0.91	-0.89	-0.96	-0.96	-0.92	-0.32	-0.72
ISO	-0.96	1	0.97	0.95	0.95	0.93	0.97	0.98	0.82	0.21	0.6
Map	-0.99	0.97	1	0.92	0.91	0.9	0.96	0.96	0.92	0.33	0.72
Mat	-0.91	0.95	0.92	1	1	1	0.99	0.99	0.71	-0.03	0.45
Max	-0.91	0.95	0.91	1	1	1	0.99	0.99	0.7	-0.04	0.43
Min	-0.89	0.93	0.9	1	1	1	0.98	0.98	0.68	-0.08	0.4
Vapor	-0.96	0.97	0.96	0.99	0.99	0.98	1	1	0.8	0.09	0.56
AET	-0.96	0.98	0.96	0.99	0.99	0.98	1	1	0.8	0.09	0.56
PWQ	-0.92	0.82	0.92	0.71	0.7	0.68	0.8	0.8	1	0.55	0.92
PDQ	-0.32	0.21	0.33	-0.03	-0.04	-0.08	0.09	0.09	0.55	1	0.56
PS	-0.72	0.6	0.72	0.45	0.43	0.4	0.56	0.56	0.92	0.56	1
